# Mid-term sustained relief from headaches after balloon angioplasty of the internal jugular veins in patients with multiple sclerosis

**DOI:** 10.1371/journal.pone.0191534

**Published:** 2018-01-23

**Authors:** Clive B. Beggs, Alessia Giaquinta, Massimiliano Veroux, Ester De Marco, Dovile Mociskyte, Pierfrancesco Veroux

**Affiliations:** 1 Institute for Sport, Physical Activity and Leisure, School of Sport, Leeds Beckett University, Leeds, United Kingdom; 2 Vascular Surgery and Organ Transplant Unit, Azienda Ospedaliero-Universitaria Policlinico, Catania, Italy; Universita degli Studi di Catania, ITALY

## Abstract

**Objectives:**

Multiple sclerosis (MS) patients frequently suffer from headaches and fatigue, and many reports have linked headaches with intracranial and/or extracranial venous obstruction. We therefore designed a study involving MS patients diagnosed with obstructive disease of internal jugular veins (IJVs), with the aim of evaluating the impact of percutaneous transluminal angioplasty (PTA) on headache and fatigue indicators.

**Methods:**

286 MS patients (175 relapsing remitting (RR), 75 secondary progressive (SP), and 36 primary progressive (PP)), diagnosed with obstructive disease of IJVs, underwent PTA of IJVs during the period 2011–2015. This included 113 headache positive patients (82 RR, 22 SP, and 9 PP) and 277 fatigue positive patients (167 RR, 74 SP, and 36 PP). *Migraine Disability Assessment* (MIDAS), and the *Fatigue Severity Scale* (FSS) were evaluated: before PTA; 3-months after PTA; and at final follow-up in 2017. Patients were evaluated with Doppler sonography of the IJVs at 1, 6 and 12 months after PTA and yearly thereafter. Non-parametric statistical analysis was performed using a combination of the Friedman test and Spearman correlation analysis.

**Results:**

With the exception of the PP patients there were significant reductions (all p < 0.001) in the MIDAS and FSS scores in the 3-month following PTA. The improvement in MIDAS score following PTA was maintained throughout the follow-up period in both the RR (p < 0.001; mean of 3.55 years) and SP (p = 0.002; mean of 3.52 years) MS cohorts. With FSS, significant improvement was only observed at 2017 follow-up in the RR patients (p < 0.001; mean of 3.37 years). In the headache-positive patients, post-PTA MIDAS score was significantly negatively correlated with the change in the blood flow score in the left (r = -0.238, p = 0.031) and right (r = -0.250, p = 0.023) IJVs in the RR patients and left IJV (r = -0.727, p = 0.026) in the PP patients. In the fatigue-positive cohort, post-PTA FSS score was also significantly negatively correlated with the change in blood flow in the right IJV in the PP patients (r = -0.423, p = 0.010). In addition, the pre and post-PTA FSS scores were significantly positively correlated in the fatigue-positive RR (r = 0.249, p = 0.001) and SP patients (r = 0.272, p = 0.019).

**Conclusions:**

The intervention of PTA was associated with a large and sustained (>3 years) reduction in MIDAS score in both RR and SP MS patients. While a similar initial post-PTA reduction in FSS score was also observed, this was not maintained in the SP and PP patients, although it remained significant at follow-up (>3 years) in the RR MS patients. This suggests that venoplasty might be a useful intervention for treating patients with persistent headaches and selected concomitant obstructive disease of the IJVs.

## Introduction

In recent years percutaneous transluminal angioplasty (PTA) of the internal jugular veins (IJVs) has been used to treat chronic cerebrospinal venous insufficiency (CCSVI), a vascular condition reportedly associated with multiple sclerosis (MS) that is characterized by constricted cerebral venous outflow [1–5]. This has resulted in many thousands of operations being undertaken worldwide, with the PTA procedure that is generally considered to be safe [6–8]. Despite this, the use of PTA to alleviate CCSVI in MS patients remains controversial [9, 10]. In particular, relatively few quality of life (QOL) studies have been performed to evaluate the clinical benefits of PTA in patients with obstructive disease of the IJVs, with discordant results. While some studies have found PTA to improve QOL indicators in MS patients [8, 11–14], others [15] appear to contradict this finding. Consequently, there is need for clarity regarding the clinical benefits of PTA.

Among the symptoms reportedly responding to PTA treatment, two candidates in particular appear to be worthy of further investigation, headache and chronic fatigue. Bavera [13] in a prospective investigation involving 366 consecutive MS patients who underwent PTA and were subsequently interviewed by an independent assessor and followed up for 4 years, found improvements respectively in 98.6% of patients with headache and in 98.5% of cases with associated chronic fatigue. This latter symptom was also investigated longitudinally by Malagoni et al [16] using two validated scales and an independent non-blinded assessor, who reported significant improvements one year after the procedure. Kostecki et al [14] also reported significant improvements in patient fatigue six months after PTA. Furthermore, there is abundant evidence linking headaches with obstruction of the cerebral venous drainage pathways [17–22], suggesting that PTA might be an effective intervention for patients suffering from persistent headaches. However, while many reports link headaches with intracranial and/or extracranial venous obstruction, there are no reports specifically investigating the relationship between obstructive disease of the IJVs and headaches. We therefore designed the repeated measures study reported here involving 286 MS patients who underwent PTA for obstructive disease of IJV, with the aim of evaluating the impact of the procedure on headache and fatigue indicators.

## Materials and methods

This was a single-center open label observational study, with data collected prospectively but analyzed retrospectively, designed to evaluate, using a standardized and operator-independent catheter venography protocol, the impact of PTA on neurological symptoms such as headache and fatigue in patients with MS. The study was unfunded, with the Italian National Health System covering all the procedure costs. The patients and investigators were not paid for their participation. The study had a specific approval by the Ethical Committee of the University Hospital of Catania for the retrospective evaluation of morphological and hemodynamic changes in internal jugular outflow before and after balloon angioplasty. All patients signed an informed consent form on which the potential risks and benefits of the study treatment were detailed. Patients were also conscious that venoplasty was not performed in order to treat MS.

All patients underwent Duplex ultrasound (DUS) of their IJVs. Patients with multiple sclerosis and a history of headaches or fatigue, a DUS stenosis >50% of both IJVs and at least 12 months post-PTA follow-up were included in the study. Patients with unilateral stenosis or patients with IJV bilateral thrombosis, previous PTA of IJV, patients with IJV muscle compression and hypoplasia of IJVs, presence of pace makers, documented severe intolerance to contrast medium and no compliance with therapy were excluded. Patients who underwent PTA during the follow-up period were also excluded from the study. IJV morphologic and hemodynamic anomalies were documented pre- and post-PTA, using in all patients a standardized, validated, operator-independent catheter venography protocol [23, 24], while PTA was performed using a standardized technique [23, 24].

From an original cohort of 364 MS patients who underwent venoplasty, 286 patients (175 relapsing remitting (RR), 75 secondary progressive (SP), and 36 primary progressive (PP)) met the inclusion criteria of this study. As such, the study population comprised 113 headache positive patients (82 RR, 22 SP, and 9 PP) and 277 fatigue positive patients (167 RR, 74 SP, and 36 PP). These two groups were not mutually exclusive, with some patients experiencing both headaches and fatigue. In all patients the *Migraine Disability Assessment* (MIDAS), and the *Fatigue Severity Scale* (FSS), both of which have been extensively validated in patients with multiple sclerosis [16, 25, 26], were evaluated: before the PTA intervention; three months after the procedure; and at the final follow-up (April 2017). The IJVs patency was assessed with DUS in all patients at 1, 6 and 12 months after the procedure and yearly thereafter. Catheter venography of the IJVs was not performed during follow-up to evaluate the venous outflow.

IJV blood flow (clearance time of contrast dye) data was also collected pre and post-intervention, by means of a methodology previously reported [23], with clearance times categorized according to severity: category 1 (normal flow; 0–2 seconds clearance time); category 2 (mild delay; 2–4 seconds clearance time); category 3 (moderate delay; 4–6 seconds clearance time); and category 4 (severe delay; >6 seconds clearance time).

### Statistical analysis

Statistical analysis was performed using the respective MIDAS and FSS scores for the patient’s pre and post-PTA. In order to facilitate meaningful statistical analysis, patients who experienced no headaches or any fatigue post-PTA were given neutral MIDAS and FSS scores of 0 and 2.8, respectively. Statistical analysis of the data was performed using ‘in-house’ algorithms written in ‘R’ (open source statistical software).

Because the data exhibited considerable heteroscedasticity, it violated the assumptions for ANOVA. Therefore, a non-parametric Friedman test was used to analyze the repeated measures data. Because this test cannot accommodate multiple factors, it was applied to each clinical sub-group separately in order to evaluate whether or not any change in status had occurred post-PTA. Pairwise post-hoc analysis was then performed using a Wilcoxon-Nemenyi-McDonald-Thompson symmetry test [27]. In addition, the relationship between change in clinical status 3 months post-PTA and IJV clearance time pre and post-PTA was evaluated using Spearman correlation analysis. For all tests p values <0.05 were deemed to be significant.

## Results

The demographic characteristics of the clinical sub-groups is presented in Table 1, which reveals that 61.2% of patients had RR MS, 26.2% has SP MS, with a further 12.6% having PP MS. Of these patients the majority (67.5%) were female. The mean duration between the PTA procedure and last follow-up appointment was 1237 days (3.39 years) for the RR patients, 1259 days (3.45 years) for the SP patients, and 1245 days (3.41 years) for the PP patients.

**Table 1:** Demographic and clinical status of multiple sclerosis (MS) patients classified by disease sub-group.

MS type	Number of subjects	Age Mean	Number of female subjects	Number of Headache Positive	Number of Fatigue Positive	Time from procedure to follow-up (days)
	N (%)	Mean (SD)	N (%)	N (%)	N (%)	Mean (SD)
RR	175 (61.2)	41.44 (9.23)	127 (72.6)	82 (46.9)	167 (95.4)	1237.4 (454.7)
SP	75 (26.2)	48.75 (8.35)	46 (61.3)	22 (29.3)	74 (98.7)	1259.1 (428.9)
PP	36 (12.6)	45.69 (9.82)	20 (55.6)	9 (25.0)	36 (100.0)	1244.9 (411.0)

**Fig 1:**
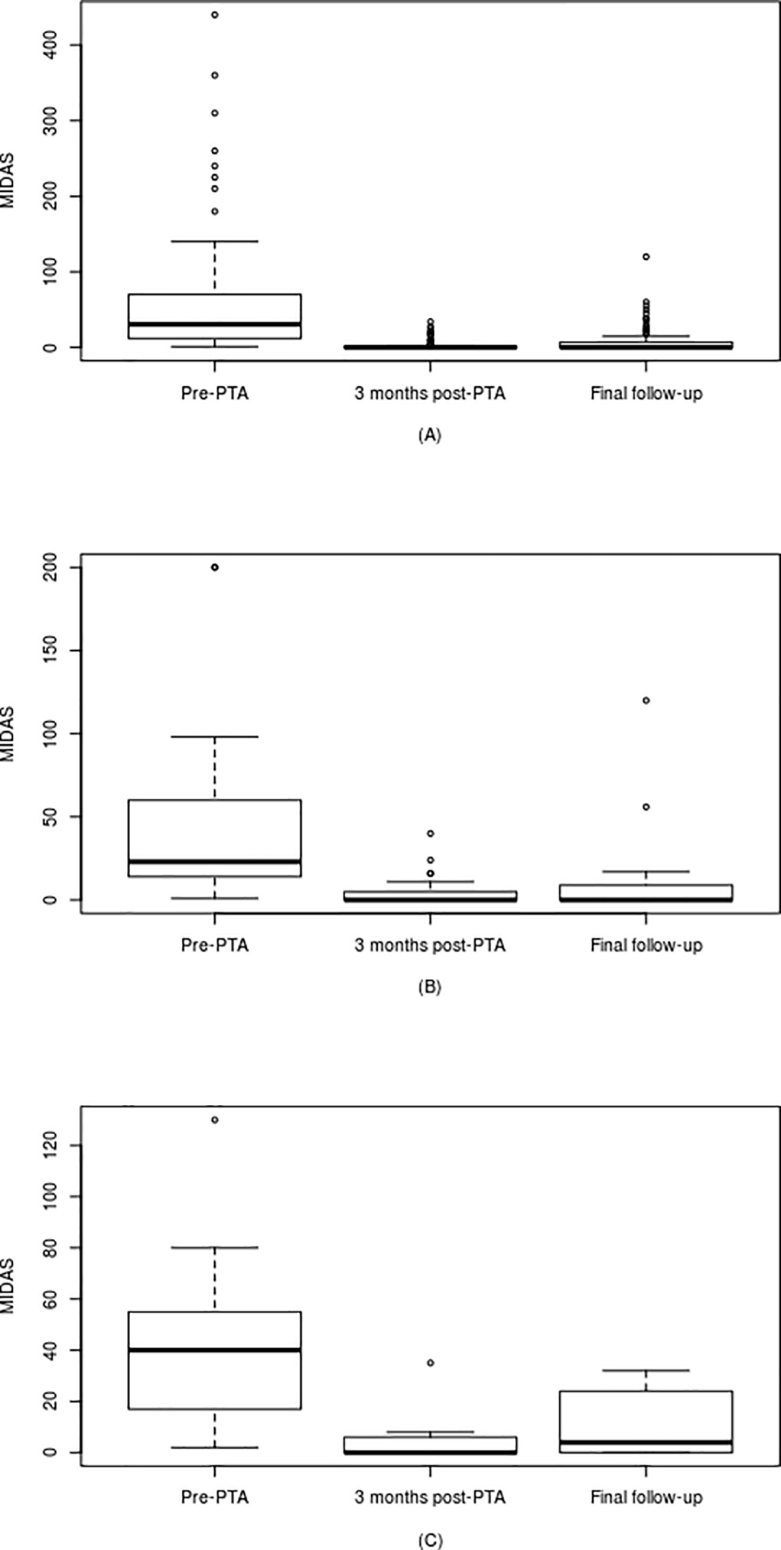
MIDAS scores at pre-PTA, 3 months after PTA and final follow-up for the: (A) relapsing remitting (n = 82); (B) secondary progressive (n = 22); and (C) primary progressive (n = 9) multiple sclerosis patients.

**Fig 2:**
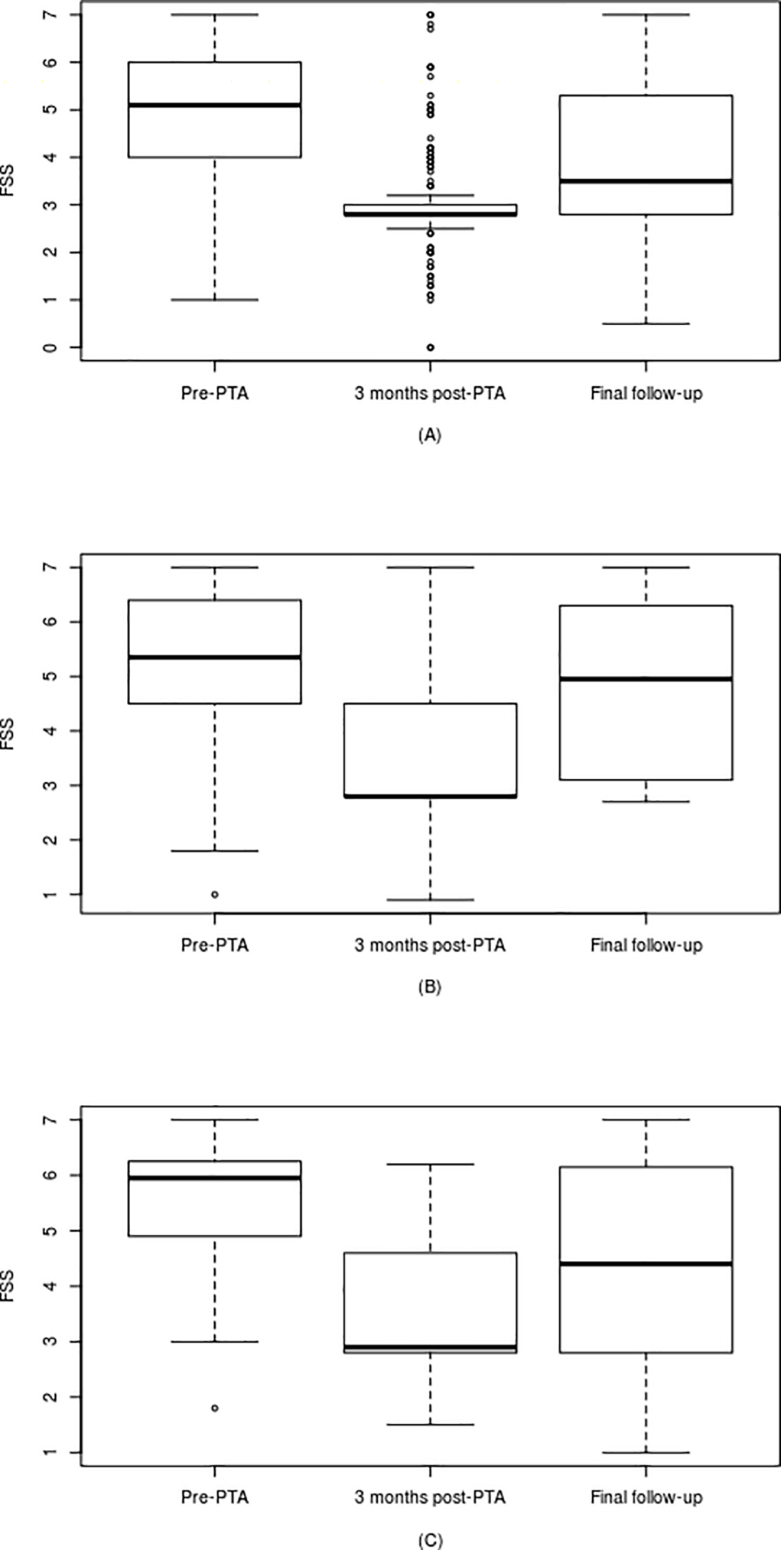
FSS scores at pre-PTA, 3 months after PTA and final follow-up for the: (A) relapsing remitting (n = 167); (B) secondary progressive (n = 74); and (C) primary progressive (n = 26) multiple sclerosis patients.

The clinical results are presented in Tables 2 and 3 and in Figs 1 and 2. From these it can be seen that with the exception of the PP MS group there were significant reductions (all p < 0.001) in the MIDAS and FSS scores in the three months following PTA. In the PP MS patients PTA was associated with a significant reduction in the FSS score shortly after PTA, but not in the MIDAS score. The improvement in MIDAS score following PTA was maintained throughout the follow-up period in both the RR (p < 0.001; mean of 3.55 years) and SP (p = 0.002; mean of 3.52 years) MS patients. With regard to the FSS score, significant improvement was only observed at 2017 follow-up in the RR group (p < 0.001; mean of 3.37 years), indicating that PTA was only associated with a sustained reduction in FSS score in this clinical group. With the exception of the headache positive PP MS patients, in all clinical sub-groups the intervention of PTA significantly increased IJV blood flow rate.

**Table 2:** Demographic and clinical statistics for headache positive multiple sclerosis patients who received PTA treatment.

MS type	No.	Time (in days) to follow-up	Variable	Pre- PTA	Pre- PTA	3 months post-PTA	3 months post-PTA	2017 follow-up	2017 follow-up	Test Statistic	Signif.	Post-hoc 1	Post-hoc 2
		Mean (sd)		(mean)	(sd)	(mean)	(sd)	(mean)	(sd)		(p value)	(p value)#	(p value)^
RR	82	1296.5 (495.1)	Age at PTA	40.86	9.42	na	na	na	na	na	na	na	na
			Right IJV flow score	2.88	0.93	2.43	0.93	na	na	5.57**	<0.001**	na	na
			Left IJV flow score	3.05	0.90	2.59	1.02	na	na	5.64**	<0.001**	na	na
			MIDAS score	61.29	82.68	2.90	7.01	8.61	18.75	108 (2)*	<0.001*	<0.001	<0.001
SP	22	1284.3 (442.6)	Age at PTA	48.55	8.04	na	na	na	na	na	na	na	na
			Right IJV flow score	3.09	0.68	2.77	0.81	na	na	2.65**	0.016**	na	na
			Left IJV flow score	3.41	0.91	3.05	0.84	na	na	2.34**	0.035**	na	na
			MIDAS score	45.50	56.87	5.23	10.33	10.45	27.46	18.9 (2)*	<0.001*	<0.001	0.002
PP	9	1271.4 (461.7)	Age at PTA	44.67	8.80	na	na	na	na	na	na	na	na
			Right IJV flow score	2.89	0.93	2.00	0.87	na	na	2.20**	0.063**	na	na
			Left IJV flow score	3.11	0.93	2.44	0.88	na	na	1.98**	0.125**	na	na
			MIDAS score	44.00	40.24	5.89	11.34	10.33	13.25	4.8 (2)*	0.091*	na	na

NB. For ease of reference the post-PTA blood flow rate scores are shown in the ‘3 months post-PTA’ columns. However, the post-PTA assessment of blood flow actually took place immediately following the procedure.

* Non-parametric repeated measures Friedman test (Friedman chi-square)

** Exact Wilcoxon-Pratt signed-rank test (Z statistic)

# Post-hoc 1: Wilcoxon-Nemenyi-McDonald-Thompson post-hoc test comparing pre and 3 months post-PTA MIDAS scores.

^ Post-hoc 2: Wilcoxon-Nemenyi-McDonald-Thompson post-hoc test comparing pre PTA and 2017 follow-up MIDAS scores.

**Table 3:** Demographic and clinical statistics for fatigue positive multiple sclerosis patients who received PTA treatment.

MS type	No.	Time (in days) to follow-up	Variable	Pre- PTA	Pre- PTA	3 months post-PTA	3 months post-PTA	2017 follow-up	2017 follow-up	Test Statistic	Signif.	Post-hoc 1	Post-hoc 2
		Mean (sd)		(mean)	(sd)	(mean)	(sd)	(mean)	(sd)		(p value)	(p value)#	(p value)^
RR	167	1447.5 (2896.0)	Age at PTA	41.34	9.25	na	na	na	na	na	na	na	na
			Right IJV flow score	2.98	0.87	2.57	0.88	na	na	7.69**	<0.001**	na	na
			Left IJV flow score	3.20	0.85	2.75	0.99	na	na	7.86**	<0.001**	na	na
			FSS score	4.93	1.51	3.08	1.14	3.95	1.59	101 (2)*	<0.001*	<0.001	<0.001
SP	74	1251.5 (426.9)	Age at PTA	48.57	8.26	na	na	na	na	na	na	na	na
			Right IJV flow score	3.16	0.83	2.69	0.91	na	na	5.57**	<0.001**	na	na
			Left IJV flow score	3.28	0.80	2.85	0.84	na	na	5.20**	<0.001**	na	na
			FSS score	5.26	1.37	3.68	1.40	4.89	1.58	47.0 (2)*	<0.001*	<0.001	0.283
PP	36	1244.9 (411.0)	Age at PTA	45.69	9.82	na	na	na	na	na	na	na	na
			Right IJV flow score	2.92	0.94	2.53	1.00	na	na	2.90**	0.005**	na	na
			Left IJV flow score	3.28	0.85	2.81	0.95	na	na	3.86**	<0.001**	na	na
			FSS score	5.53	1.22	3.61	1.13	4.47	1.88	22.8 (2)*	<0.001*	<0.001	0.163

NB. For ease of reference the post-PTA blood flow rate scores are shown in the ‘3 months post-PTA’ columns. However, the post-PTA assessment of blood flow actually took place immediately following the procedure.

* Non-parametric repeated measures Friedman test (Friedman chi-square)

** Exact Wilcoxon-Pratt signed-rank test (Z statistic)

# Post-hoc 1: Wilcoxon-Nemenyi-McDonald-Thompson post-hoc test comparing pre and 3 months post-PTA FSS scores.

^ Post-hoc 2: Wilcoxon-Nemenyi-McDonald-Thompson post-hoc test comparing pre PTA and 2017 follow-up FSS scores.

The correlation results are presented in Tables 4 and 5. These reveal a mixed picture. With regard to headaches, the post-PTA MIDAS score was significantly negatively correlated with the change in the blood flow score in the left IJV in the RR (r = -0.238, p = 0.031) and PP (r = -0.727, p = 0.026) MS patients, but not in the SP patients. In the RR patients the post-PTA MIDAS score was also significantly negatively correlated with the change in the blood flow score in the right IJV (r = -0.250, p = 0.023). By contrast, in the fatigue positive cohort, the post-PTA FSS score was significantly positively correlated with the pre-PTA FSS score in the RR (r = 0.249, p = 0.001) and SP MS patients (r = 0.272, p = 0.019). The post-PTA FSS score was also significantly negatively correlated with the change in the flow score in the right IJV in the PP patients (r = -0.423, p = 0.010). All IJVs were patent at DUS examination and at all subsequent follow-up sessions.

**Table 4:** Relationship between 3 months post-PTA MIDAS score and, pre-PTA MIDAS score, and right and left IJV flow score differences for the headache positive multiple sclerosis patients.

MS type	No.	Relationship	Correlation: r value (p value)*
RR	82	Post-PTA MIDAS score & Pre-PTA MIDAS score	-0.135 (0.225)
		Post-PTA MIDAS score & Right IJV flow score difference	-0.250 (0.023)
		Post-PTA MIDAS score & Left IJV flow score difference	-0.238 (0.031)
SP	22	Post-PTA MIDAS score & Pre-PTA MIDAS score	0.003 (0.988)
		Post-PTA MIDAS score & Right IJV flow score difference	0.047 (0.837)
		Post-PTA MIDAS score & Left IJV flow score difference	0.040 (0.860)
PP	9	Post-PTA MIDAS score & Pre-PTA MIDAS score	-0.651 (0.058)
		Post-PTA MIDAS score & Right IJV flow score difference	-0.194 (0.616)
		Post-PTA MIDAS score & Left IJV flow score difference	-0.727 (0.026)

* r value calculated using Spearman correlation test.

**Table 5:** Relationship between 3 months post-PTA FSS score and, pre-PTA FSS score, and right and left IJV flow score differences for the fatigue positive multiple sclerosis patients.

MS type	No.	Relationship	Correlation: r value (p value)*
RR	167	Post-PTA FSS score & Pre-PTA FSS score	0.249 (0.001)
		Post-PTA FSS score & Right IJV flow score difference	0.129 (0.097)
		Post-PTA FSS score & Left IJV flow score difference	-0.049 (0.530)
SP	74	Post-PTA FSS score & Pre-PTA FSS score	0.272 (0.019)
		Post-PTA FSS score & Right IJV flow score difference	0.153 (0.195)
		Post-PTA FSS score & Left IJV flow score difference	-0.144 (0.222)
PP	36	Post-PTA FSS score & Pre-PTA FSS score	0.108 (0.532)
		Post-PTA FSS score & Right IJV flow score difference	-0.423 (0.010)
		Post-PTA FSS score & Left IJV flow score difference	0.005 (0.975)

* r value calculated using Spearman correlation test.

## Discussion

Headache is a common, complex and multifactorial neurological symptom which significantly reduces QOL [28, 29], with a vast proportion of patients reported to be poor responders to available therapies [30, 31]. While the pathophysiology underlying headaches is poorly understood, there is strong evidence linking the condition with obstruction of the cerebral venous drainage system [17–22]. Indeed, several studies have shown IJV compression to aggravate headache intensity in patients with migraine [32, 33], suggesting that venous hypertension, caused by increased venous blood retention in the cortical vessels, might be an influential factor in the pathophysiology of headache [34]. It is thought that elevated cerebral venous pressure can result in a dilated dural sinuses and cerebral veins, and that mechanical stimulation of these pain-sensitive vessel structures might lead to headaches [34].

The fact that headaches intensify when the IJVs are compressed [32, 33], suggests that a similar phenomenon may occur when cerebral venous drainage is constricted. Being thin walled floppy vessels, the cortical veins readily accumulate blood and greatly expand when the IJVs are compressed [35]. As such, there is good reason to believe that any significant stenosis of the extracranial cerebral drainage pathways might have a similar effect, albeit of lesser magnitude, resulting in raised intracranial venous pressure [36]. Gadda et al [37] in a computational study, calculated that obstruction of both IJVs would cause the venous sinus pressure to increase by >7 mmHg when in the supine position. A similar finding was recently reported by Tessari et al [38] who calculated significant increases in pressure in the superior and inferior petrosal sinuses arising from occlusion of the IJVs due to faulty valves. In addition, cervical plethysmography has shown that in MS patients diagnosed with CCSVI the hydraulic resistance of the extracranial cerebral venous drainage pathways is increased by 63.5% [39], suggesting the presence of raised venous sinus pressure in this patient group [36]. These findings suggest that intracranial venous hypertension might be a feature of this neurological condition. Evidence supporting this comes from two recent studies by Bateman et al, who found that MS patients exhibited: (i) a 35% reduction in arteriovenous delay, indicative of reduced intracranial compliance [40]; and (ii) a 16% increase in superior sagittal sinus cross-sectional area [41]. During systole, venous blood stored in the cortical veins during diastole is freely discharged from the cranium via the sinuses [42]. In doing so, the cortical veins that traverse the sub-arachnoid space interact with the cerebrospinal fluid (CSF) [42], imparting a functional compliance to the intracranial space [43, 44]. Because the functional compliance of the cortical bridging veins relies on their ability to empty during systole, any constriction that inhibits the discharge of venous blood from the cranium has the potential to reduce intracranial compliance–something that might result in a general stiffening of the brain parenchyma as Hatt et al [45] observed when they compressed the IJVs in healthy subjects. If raised intracranial venous pressure is a feature of MS, then this would be consistent with the findings of Bateman et al [40, 41], and might help to explain why MS patients are so prone to migraine headaches [46, 47].

The principal finding of our study that PTA is associated with a large and sustained reduction in MIDAS score in both RR and SP patients appears to be consistent with thinking that venous hypertension is a contributory factor to headaches in MS patients. Using PTA to restore IJVs flow should in theory reduce the hydraulic resistance of the extracranial venous pathways back to the heart and thus help to minimize any retrograde hypertension that may be present [11]. The clinical benefits of PTA with regard to headaches are clearly evident in Fig 1, where an 86% reduction (p < 0.001) in mean MIDAS score can be observed in the RR patients at follow-up (3.5 years after PTA), with a similar (77%) reduction (p = 0.002) after 3.5 years (follow-up) observed in the SP cohort. A similar trend was also observed in the PP patients, although this did not reach significance. As such this suggests that PTA is capable of providing sustained relief from headaches in patients with MS.

In addition to the QOL indicator data, we also collected pre- and post-intervention IJV blood flow data from both sides of the neck. For all clinical sub-groups the intervention of PTA increased IJV blood flow, with this increase reaching significance in all but the headache positive PP MS patients, something that may be more indicative of the low number of PP patients involved (n = 9), rather than any physiological differences associated with this particular sub-group. Indeed, with respect to IJV flow, post-hoc analysis (Mann-Whitney U-test–results not shown) revealed little difference between any of the headache-positive clinical sub-groups, with the only exception being for the right IJV where post-PTA blood flow was significantly lower in the PP group compared with the SP MS patients (p = 0.040). As such, our study suggests that the impact of venoplasty on IJV blood flow was broadly similar for all the clinical sub-groups.

With respect to pre- and post-intervention IJV blood flow, we assumed that the flow data would support any QOL improvements observed. However, although we found that PTA significantly improved IJV drainage in all the clinical sub-groups (except the PP MS patients in the headache positive cohort), these improvements were not necessarily associated with any improvement in the MIDAS and FFS scores 3 months after the intervention, which was the first follow-up opportunity to assess the QOL metrics. From the correlation analysis results presented in Tables 4 and 5 it can be seen that a complex picture emerges. Table 4 reveals that the post-PTA MIDAS score at 3 months was significantly negatively correlated with increased blood flow in both IJVs in the RR patients, with the effect size being small to medium, whereas, little or no effect was observed in the other clinical sub-groups. Although a strong negative correlation was also observed between the post-PTA MIDAS score and change in blood flow in the left IJV in the PP MS patients, it should be remembered that only nine patients were included in this group, with the result that this observation should be treated with caution. In addition, it should be noted that of necessity the QOL scores were recorded 3 months after the pre- and post-PTA blood flow rates were measured. As such, a time delay was introduced which may have acted as a confounding factor. Having said this, the fact that we found significant correlations between blood flow and MIDAS score in some patient groups 3 months post-PTA, means that we cannot exclude the possibility that improved blood flow arising from the intervention may also have contributed to the reduction in MIDAS score. Chronic hypoperfusion of the brain has been widely reported in MS patients [48–51], and migraine headaches have been associated with cortical hypoperfusion [52–54]. So, it may be that the marked post-PTA reduction in MIDAS score that we observed in this study was attributable to a combination or reduced hypertension in the venous sinuses and improved perfusion of the cortex.

In comparison to the headache results, the picture regarding venoplasty and fatigue is much less clear. In all three patient groups, the intervention of PTA resulted in dramatic reductions in FSS score immediately following the procedure. However, over the next three to four years this beneficial effect weakened, so that only in the RR group was a significant (20%) reduction in FSS score (p<0.001) still observed at final follow-up. While dramatic reductions in both FSS and MIDAS score were observed immediately following venoplasty, it is noticeable that the ‘rebound’ effect (Fig 2) applied only to FSS score and was not observed with MIDAS score. While it might be tempting to attribute this rebound to a wearing-off of a possible placebo effect, it is difficult to explain why it should only occur with regard to fatigue and not headaches. Furthermore, the placebo effect cannot explain why a significant clinical improvement in FSS score was still observed at follow-up in the RR MS patients, more than 3 years after the intervention. It therefore appears likely that the reported FSS results represent a real effect that, although present directly after the PTA procedure, diminished with time. With regard to this, the correlation results in Table 5 suggest that the post-PTA FSS scores were primarily associated with the pre-PTA FSS scores in the RR and SP patients, rather than with any increase in IJV blood flow. Indeed, in both these clinical sub-groups the relationship was a significant positive correlation with a small to medium effect size. As such, this suggests that the headaches and fatigue experienced by MS patients may arise from different pathophysiological processes, which might respond to PTA in different ways. While any link between fatigue and constricted cerebral venous outflow has not been established, it is known that the lateral ventricles are enlarged in patients with chronic fatigue syndrome [55] and in MS patients with greater levels of cognitive fatigue [56]. Given that CSF pulsatility in the Aqueduct of Sylvius has been shown to significantly increase in MS patients [57, 58], and that this phenomenon is linked with constricted cerebral venous outflow [42, 45, 59, 60], it may be that in our study, PTA influenced the FSS score by reducing the size of the lateral ventricles. Indeed, the surgical restoration of jugular flow has been shown to decrease lateral ventricle volume and improve cerebral perfusion in RR MS patients, but not in SP patients [61].

Although our study yielded valuable insights into the clinical benefits of PTA as an intervention for treating symptoms associated with MS, it also raises intriguing questions as to why the initial dramatic improvements in FSS score observed post-PTA were not maintained at follow-up, despite the fact that reduced MIDAS scores were sustained. One limitation of the study was that catheter venography assessment of IJV outflow was not performed at the scheduled follow-up appointments, with IJV patency evaluated using only DUS. Although there are no validated comparable methods to angiography (including DUS), with which to evaluate the hemodynamic results of PTA in the long-term, performing venous angiography to evaluate hemodynamic performance in the long-term may be unreliable, particularly in stable patients. As a result, DUS was used in all patients only to produce a qualitative assessment of the IJV flow. As a consequence, this study was not able to corroborate whether this FSS rebound effect was due to impaired jugular flow, restenosis, as reported by other authors [8, 11, 14] or some other unknown effect. Whilst further investigations will be needed to investigate precisely why the observed FSS rebound effect occurs, we are conscious that randomized controlled trials investigating this issue may only become feasible once new venous-oriented devices have been developed that improve the technical success of venous angioplasty, ensuring that improved IJV flow is maintained over a long period of time. In addition, we recommend that future studies should investigate the role of the Azygos and lumbar vein systems in the pathophysiology of headache and fatigue, as stenosis of these veins might also be influential.

## Conclusions

In summary, the principal finding of the study is that with regard to headaches, the intervention of PTA appears to be associated with a large and sustained (>3 years) reduction in MIDAS score in both RR and SP MS patients. While a similar initial post-PTA reduction in FSS score was also observed, this was not maintained in the SP and PP patients groups, although it remained significant at follow-up (>3 years) in the RR MS patients, despite the effect being greatly reduced. As such, our findings suggest that PTA might be a useful intervention for treating MS patients with persistent headaches and selected obstructive disease of the IJVs.

## Supporting information

S1 File Data used in the study. (XLS)
